# Studying T Cell Responses to Hepatotropic Viruses in the Liver Microenvironment

**DOI:** 10.3390/vaccines11030681

**Published:** 2023-03-17

**Authors:** Jarrett Lopez-Scarim, Shashank Manohar Nambiar, Eva Billerbeck

**Affiliations:** Division of Hepatology, Department of Medicine and Department of Microbiology and Immunology, Albert Einstein College of Medicine, Bronx, NY 10461, USA

**Keywords:** T cells, hepatotropic virus infection, liver immunology, hepatitis A–E, animal models

## Abstract

T cells play an important role in the clearance of hepatotropic viruses but may also cause liver injury and contribute to disease progression in chronic hepatitis B and C virus infections which affect millions of people worldwide. The liver provides a unique microenvironment of immunological tolerance and hepatic immune regulation can modulate the functional properties of T cell subsets and influence the outcome of a virus infection. Extensive research over the last years has advanced our understanding of hepatic conventional CD4+ and CD8+ T cells and unconventional T cell subsets and their functions in the liver environment during acute and chronic viral infections. The recent development of new small animal models and technological advances should further increase our knowledge of hepatic immunological mechanisms. Here we provide an overview of the existing models to study hepatic T cells and review the current knowledge about the distinct roles of heterogeneous T cell populations during acute and chronic viral hepatitis.

## 1. Introduction

The human liver is the target organ of five hepatotropic viruses, specifically hepatitis viruses A–E. Infection with hepatitis A (HAV) or E (HEV) virus occurs via the fecal-oral route and predominantly results in acute hepatitis and viral clearance [1,2,3]. In contrast, blood-borne hepatitis B (HBV) or C (HCV) virus cause an acute-resolving infection only in a minority of patients, while the majority of those infected will develop a chronic infection [4,5,6,7]. Hepatitis D virus (HDV) is a satellite virus and exists only as coinfection with HBV [8,9]. Chronic viral hepatitis is a major global health problem as it can lead to progressive liver disease, including fibrosis, cirrhosis, and hepatocellular carcinoma (HCC) [10]. Despite the availability of an effective prophylactic HBV vaccine, approximately 290 million people are chronically infected with HBV worldwide. Curative treatment for HBV does not exist [5]. In contrast, chronic HCV, which affects approximately 58 million people worldwide, can be cured with highly effective direct acting antivirals (DAA) but a prophylactic vaccine is lacking [4,11].

At the pathological level, acute-resolving hepatitis is primarily characterized by hepatic infiltration of immune cells, elevated levels of alanine transaminase (ALT), hepatocyte and bile duct damage, accompanied by cholestasis and jaundice [1,12]. Acute-resolving infection occurs over a period of a few weeks to a few months and the pathological effects on the liver are typically mild and temporary with low levels of hepatocytic apoptosis and fibrosis. In contrast, the pathological features of chronic viral hepatitis include persistent immune cell activation, inflammation, and liver damage that can eventually progress to cirrhosis and HCC development [10,12].

T cell subsets play a central role in the clearance of hepatic virus infections, but they can also mediate liver injury during acute hepatitis and sustain inflammation and contribute to disease progression during chronic infection [13,14,15]. A detailed understanding of antiviral, pathological, and immune-regulatory T cell mechanisms in the hepatic microenvironment is a prerequisite for the development of T cell-based vaccine strategies for hepatic viruses. In fact, the liver has unique immunological features that are directly linked to its function as a major metabolic organ of the body [16]. A distinct composition of innate and adaptive immune cell subsets mediates a tolerogenic immune response to constant exposure to gut-derived microbial antigens [16]. This tolerogenic state is overcome during viral infection and, ideally, a tightly regulated immune response which results in acute viral clearance with limited tissue pathology is induced. Studying the hepatic immune mechanisms which result in viral clearance versus persistence and chronic inflammation is complicated by limited access to human liver tissue, especially during acute infection, and the limited availability of immune-competent animal models [17,18,19,20]. Despite these difficulties, our understanding of hepatic antiviral T cell immunity is expanding, facilitated by the development of new animal models and improved techniques such as high-dimensional flow cytometry and single-cell RNA-sequencing (sc-RNA-seq.) which allow an in-depth analysis of rare human tissue.

Here we will first summarize the current models used to study hepatic T cell responses to hepatitis A–E and then review present knowledge derived from human and animal studies about the function of different hepatic T cells populations, including CD4+ and CD8+ T cells, regulatory T cells, and unconventional T cell subsets during acute and chronic virus infections.

## 2. Animal Models of the Hepatic T Cell Response to Hepatitis A–E Infection

Hepatitis viruses A, B, C, and D naturally have an exclusive tropism for the human liver, while HEV is a zoonotic virus [17,18,19,20,21]. Studies on T cell subsets obtained from the peripheral blood or, when available, liver tissue samples, of infected patients critically contribute to the expanding knowledge of antiviral T cell immunity to hepatic virus infections. While these correlative studies with human samples are most relevant, identifying immune mechanisms in the liver microenvironment or assessing vaccine strategies usually requires experimental animal models.

Different species of non-human primates (NHPs) can be experimentally infected with all five human hepatitis viruses, with HCV having the narrowest host range to only humans and chimpanzees [22,23]. NHPs, especially the chimpanzee, have greatly contributed to the discovery of human hepatitis viruses and the understanding of their viral life cycle, pathogenesis, immunity, and vaccine development. However, inaccessibility and ethical concerns have ended the use of the chimpanzee in viral hepatitis research [22].

Immune-competent laboratory mice, the most tractable model for immunological studies, are not readily susceptible to any human hepatitis virus. Yet, over the last several decades, numerous immune-compromised and competent small animal models have been developed that allow the study of various aspects of hepatitis A–E biology in vivo. Comprehensive reviews summarizing all animal models for the five human hepatitis viruses have been published recently [17,18,19,20,21]. In the following section, we will only highlight small animal models that allow the study of hepatic T cell immunity (rodent models are summarized in Supplementary Table S1).

### 2.1. Immune-Competent Non-Infection Mouse Models

Mice can generally not be infected with HCV, HBV, or HDV because mouse hepatocytes lack the viral entry factors for these viruses [21,24,25]. To bypass this roadblock, immune-competent transgenic mice expressing single HBV proteins or the whole HBV genome in hepatocytes have been generated [26,27]. Mice transgenic for the entire genome can produce infectious HBV viral particles in mouse hepatocytes and show viremia in the peripheral blood [28]. Due to the transgenic nature of this model, mice are immune-tolerant to HBV and do not show liver pathology [28]. However, adoptive transfer of immune cells, such as T cell receptor (TCR)-transgenic T cells specific for HBV epitopes from donor mice, can induce HBV-specific immune responses [29]. HBV transgenic mice in combination with adoptive T cell transfer, have been used in numerous studies to investigate HBV-specific T cell mechanisms in the liver [30].

Another approach to induce HBV replication in immune-competent mice is the delivery of HBV genomes into the liver using viral vectors (AdV, AVV) or hydrodynamic tail vein delivery of plasmids [31,32,33,34]. These methods can lead to short-term or persistent HBV replication in mice, result in the induction of an antiviral immune response, and can lead to liver fibrosis development [32,35,36]. Interestingly, mice with viral vector mediated HBV replication develop signs of immunological tolerance to HBV similar to chronically HBV infected patients. As a result, this model might be useful for studying mechanisms of HBV immune tolerance [35,37]. The techniques used in this model can also be applied to the study of HDV, as shown in a recent study which established an immune-competent HDV mouse model using AAV-mediated delivery of HBV and HDV [38]. Liver damage and a strong innate immune response amplified by adaptive immunity were observed in this model.

HBV transgenic and transduction models enable studies on hepatic antiviral T cell immunity, but the incomplete viral life cycle may induce a different immune response than natural infection. In addition, in vector-based systems the influence of the viral vectors on immune responses and disease development in these models needs to be taken into consideration.

### 2.2. Immune-Compromised Infection Mouse Models

HAV is the only human hepatitis virus that can readily infect laboratory mice; however, these mice need to be immune compromised to some degree [39]. It was found that type I interferon receptor deficient (Ifnar1^−/−^) mice and MAVS (mitochondrial antiviral-signaling protein)^−/−^ mice are susceptible to HAV. Further investigation suggested this susceptibility is the result of the ability of HAV to cleave human but not mouse MAVS and thus blunt the human type I interferon response. This work indicates that MAVS is the determinant of HAV’s host range [39]. Ifnar1^−/−^ mice show HAV pathogenesis similar to humans, with acute liver injury but no mortality and hepatic immune cell infiltration. This mouse model has been used to study T cell responses during HAV infection [40]. However, the lack of type I interferon signaling likely impairs the T cell response and further improvements of this model to make it more suitable for the study of HAV immunity are necessary.

### 2.3. Surrogate Models

Another approach to potentially develop immune-competent models for hepatitis A–E is to explore genetically related viruses that naturally infect other species.

HCV belongs to the family of *Flaviviridae*, genus hepacivirus, and for decades only one other hepacivirus was known: Georg Baker virus (GBV), which infects New World monkeys [41]. However, since 2013 new hepaciviruses have been discovered in several species including horses, bats, cows, sharks, and rodents [41]. The genetic similarity between these animal hepaciviruses and HCV varies, with the virus discovered in horses (non-primate hepacivirus, NPHV) being the closest relative to HCV to date [42]. The discovery of rodent hepaciviruses (RHV) was an important step in developing small animal models for studying antiviral immunity [43,44]. In fact, it was shown that variants of rodent hepaciviruses isolated from Norway rats (RHV-nr-1 or NrHV) could infect immune-competent laboratory rats and mice and establish a high-titer hepatotropic infection [45,46]. Rats develop a persistent infection while standard laboratory mice (C57BL/6 and Balb/c) clear the virus within 3–5 weeks post-infection [45,46]. Viral clearance of NrHV in mice is CD4+ and CD8+ T cell dependent. Transient CD4+ T cell depletion prior to infection in mice results in viral chronicity [45]. Both rats and mice show a significant activation of hepatic immune responses associated with acute or chronic liver injury. These rodent surrogate models of HCV show similarities to human infection and allow mechanistic studies of antiviral immunity and the assessment of HCV vaccine strategies [47]. A caveat of the models is the significant genetic difference (70% amino acid divergence) between rodent hepaciviruses and HCV [41] which may impact the translation of findings from the rodent models to human disease.

HEV is a zoonotic virus of the *Hepeviridae* family and naturally infects several species, including rabbits and pigs [20]. Cross-species transmission of specific HEV genotypes (genotypes 3 and 4) is observed between humans, pigs, and rabbits and these animals have been used as natural host infection models for HEV [20,48,49]. Rabbits have been used in HEV vaccine studies [49] but, like pigs, have played a limited role in the study of hepatic T cell immunity. Since rodent models are better suited for studies of T cell immunity, developing an HEV mouse model would be extremely useful. Interestingly, unlike RHV, a rat homolog of HEV discovered in several wild rat species only establishes robust infection in immune-compromised but not competent laboratory rats [50]. Thus, the development of a rodent surrogate model for HEV seems more challenging than for HCV.

HBV is a member of the *Hepadnaviridae* family and HBV-related animal hepadnaviruses have been discovered in several species including woodchucks, ducks, and woolly monkeys [17]. Woodchuck hepatitis virus (WHBV) infection in woodchucks shares significant similarities with HBV infection in humans, such as a similar course of infection, immune responses, and liver disease progression with HCC development [51,52]. Thus, despite difficulties in working with these animals and the limited availability of immunological reagents, the woodchuck model has been used for numerous studies on HBV-related immunity, including T cell responses and pathogenesis [53].

## 3. The Hepatic Immune Environment

Before understanding how T cell subsets respond to various hepatic viral infections, it is important to become acquainted with the structure and cellular components of the hepatic environment.

### 3.1. Liver Structure and Hepatic Cells

**Figure 1:**
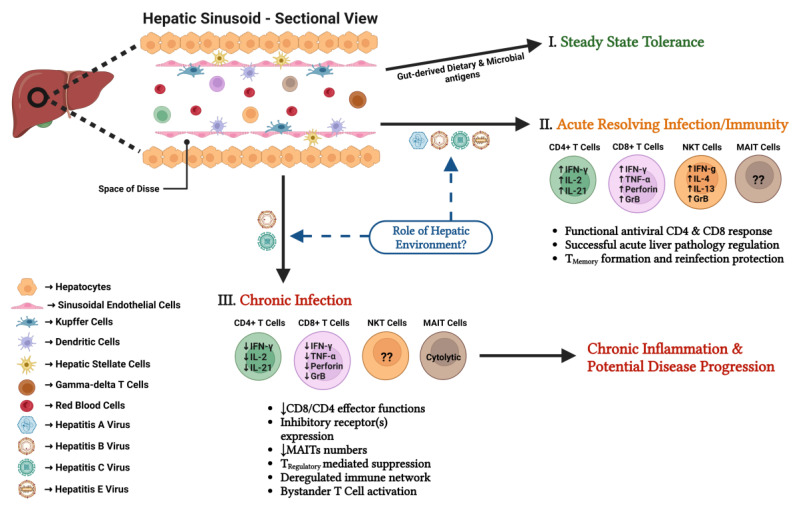
T cell immunity to viral infections in the liver. (**I**) Under steady state conditions the healthy liver is in a state of immune tolerance towards gut-derived dietary and microbial antigens. (**II**) Infection with hepatitis A, B, C, or E virus can result in an acute-resolving infection generally characterized by a functional antiviral T cell response, regulation of tissue pathology, and formation of (partial) protective immunity. (**III**) Infection with hepatitis B and C virus can lead to a chronic infection characterized by T cell dysfunction, dysregulated immune networks, chronic inflammation, and potentially progressive liver disease. The role of the specific hepatic immune environment in mediating viral clearance (**II**) or persistence (**III**) of the different hepatic viruses is not completely understood. Upward pointing arrow: increase; downward pointing arrow: decrease.

The liver is well known for being the major organ for metabolism, detoxification, and homeostatic maintenance. Structurally, the liver is formed by a tightly packed arrangement of hexagonally shaped, columnar anatomical units known as the hepatic lobule, which consists of three components: (1) portal triad, (2) parenchymal region, and (3) central vein [54]. Portal triads are located at the “vertices” of the hepatic lobule and are composed of a bundle of three vessels, including the hepatic artery, the portal vein and the bile duct [55]. Oxygen- and nutrient-rich blood drains into the hepatic lobule via the hepatic artery and portal vein, while the bile duct transports bile to the gall bladder. The bile duct is formed by biliary epithelial cells (BECs) [55]. The parenchymal region represents the lobular space between the outer portal triads and the medially located central vein. This region is primarily occupied by hepatocytes, the functional cells of the liver, which are arranged into rows along the portal triad-to-central vein axis. The parenchymal region also consists of capillary-like channels, called sinusoids, located between the rows of hepatocytes (Figure 1). Hepatic sinusoids are formed by liver sinusoidal endothelial cells (LSECs) [56,57]. The positioning of sinusoids between hepatocyte rows generates a gap (space) between the two components and is called the ‘Space of Dissé’ [56]. This space is populated by hepatic stellate cells (HSC), which store fat, vitamin A, and various retinoids [58]. The central vein collects the “processed” blood and directs it out of the liver through the hepatic vein. The structural design of the liver ensures that the blood is distributed uniformly throughout the organ and that the blood flow rate is modulated to facilitate the efficient exchange of materials into and out of the cells [59].

### 3.2. Hepatic Immune Cell Subsets

The specialized hepatic microenvironment harbors a unique composition of innate and adaptive immune cells [60] (Figure 1). The liver contains a large population of resident macrophages, Kupffer cells (KCs), which comprise ~15% of the total hepatic cells. The primary function of KCs is to phagocytose and clear all extraneous materials (such as gut-derived organic and xenobiotic particulates, cellular debris from gut fauna and tissues, and free-floating serum biomolecular complexes) that failed to be processed by hepatocytes and LSECs. KCs are located within the lumen of hepatic sinusoids, in close contact with LSECs. While they are distributed throughout the hepatic lobule, the density of KCs around the portal triad is preferentially greater. In addition to KCs, the liver contains resident hepatic dendritic cell subsets (DCs). These professional antigen-presenting cells (APCs) are located in three specific regions of the liver: below the capsular membrane and around both the periportal- and pericentral-zones of the hepatic lobules [60,61].

T cell subsets are important for immune surveillance of the liver in case of pathogenic infection. Conventional CD4+ and CD8+ T cells are not tissue-resident and constantly migrate between the liver and the secondary lymphoid organs [62]. During an infection, antigen activated naïve CD4+ T cells differentiate into helper T cell (Th) subsets (Th1, Th2, T follicular helper (Tfh), Th17) that secrete various cytokines and facilitate a pro-inflammatory response [63]. CD4+ regulatory T cells (Treg) can facilitate the immunosuppression of pro-inflammatory and cytotoxic immune cells [64]. Naïve CD8+ T cells develop into cytotoxic T cells post cognate antigen recognition and are tasked with cytolyzing their target cells [62]. Additionally, through the course of an infection, both CD4+ and CD8+ effector T cells also differentiate into memory T cells which migrate and settle into different lymphoid organs (central memory T cells) or are retained in the liver (tissue resident memory T cells (Trm)) and their function is to protect an organism from reinfection [62].

The liver is enriched in subsets of tissue-resident innate-like unconventional T cells (UTCs) [65,66]. The innate-like functionality of UTCs is an outcome of their specialized semi-invariant or invariant TCR and their ability to become activated in TCR dependent or independent mechanisms. Upon activation, UTCs rapidly produce pro- or anti-inflammatory cytokines and show a cell subset differentiation similar to Th cells. UTCs are a heterogenous group comprising three distinct cell types, namely gamma-delta T cells (γδT), mucosal-associated invariant T cells (MAIT), and natural killer T cells (NKT) [65,66]. Recent research has shown significant functional overlap between different groups of UTCs despite distinct TCR expression [67].

γδT cells are named after their TCR, which is made of a γ- and δ-chain [68]. Different γδ TCRs recognize specific sets of antigens that are not clearly defined yet [69].

MAIT cells are characterized by their invariant αβ TCR; Vα7.2-Jα33 and Vα19-Jα33 in humans and mice, respectively [66]. These cells reside in various mammalian organs, including the liver; however, their percent composition differs dramatically between different species. They detect a variety of mostly bacterial and fungal antigens presented by the MHC class 1-like protein MR1 [65,66].

NKT cells are comprised of two cell subtypes, namely, type 1 NKTs (or invariant NKT (iNKT)) and type 2 NKT cells, which are also distinguished based on their TCRs [70]. TCRs of type 1 NKT cells comprise an invariant α-chain, Vα14-Jα18, and Vα24-Jα18 in mice and humans, respectively, which bind to a small repertoire of β-chains. Type 2 NKT cells, on the other hand, display a diverse TCR array. NKT cells are well known for recognizing a range of glycolipid antigens presented on CD1d [70].

### 3.3. Liver Immune Tolerance in the Steady State

The liver consistently receives blood that is rich in nutrients and a variety of molecular and microbial antigenic materials. However, given such a situation, the liver is still able to efficiently carry out its functions without eliciting overt immunological responses. This observation highlights two important properties of the liver: (1) its ability to distinguish between antigenic materials that are innocuous versus those which are harmful/pathogenic; and (2) its proclivity to maintain an immuno-tolerogenic environment.

In order to maintain a tolerogenic hepatic immune environment, the liver employs a variety of strategies. One important strategy is to suppress the immunogenic activity of T cells during homeostatic conditions. These mechanisms have been reviewed elsewhere [16,60]. Overall, naïve T cell immunogenic activity has been shown to be suppressed by hepatocytes, KCs, DCs, LSECs, and HSCs. DCs and KCs induce T cell tolerance and Treg formation by releasing various cytokines and molecules such as IL10, TGF-β, IDO (indoleamine 2,3-dioxygenase), and prostaglandins E2. CD4+ and CD8+ T cell priming by LSECs further elicits tolerant T cells and Tregs [16,60]. In addition, the unique architecture of the liver allows for CD8+ T cell priming directly by hepatocytes. When naïve CD8+ T cells enter the hepatic sinusoids, their rate of movement reduces drastically due to the decreased sinusoidal pressure and blood flow. The slow rate of movement, coupled with the fenestrated (porous) nature of the sinusoids, greatly increases the probability of naïve T cells contacting hepatocytes, presenting their cognate antigens. T cell priming by hepatocytes leads to T cell dysfunction or cell death [62].

## 4. Hepatic T Cell Subsets during Acute-Resolving Viral Hepatitis

The quality of the antiviral immune responses mounted during acute infection determines the outcome of viral infections in the hepatic microenvironment. Extensive research with human patients and animal models has established that diverse T cell subsets are central mediators of viral elimination but also of liver pathology and immune regulation during acute infection (Figure 1).

### 4.1. CD8+ T Cells

Animal models have been instrumental in directly showing the major role of CD8+ T cells in the clearance of hepatic viral infections. Early studies in chimpanzees have shown CD8+ T cells to be critical for the clearance of both HBV and HCV [71,72]. A recently developed mouse model of an HCV-related rodent hepacivirus (NrHV) further supported these results. Mice cannot clear NrHV infection when CD8+ T cells are depleted [45]. While there is strong support for the essential role of these cells in HCV and HBV, studies with other hepatic viruses have shown a less prominent role. HAV infection in chimpanzees elicits a CD8+ T cell response, but the response is delayed and weak and not linked to viral clearance [73]. An examination of HEV infection in rhesus macaques has shown that CD8+ T cell depletion delays, but does not prevent, viral clearance [74]. Overall, these animal models suggest that CD8+ T cells play an important but varying role in the clearance of hepatic viral infections.

Given the important role of CD8+ T cells in HCV and HBV clearance, an in-depth examination of the CD8+ T cell response in both infected patients and animal models over the last decades has given more profound insights into the components of a successful CD8+ T cell effector response (reviewed [14,75,76]). Overall, studies from acutely infected patients, mostly examining peripheral blood-derived virus-specific CD8+ T cells, have shown that viral clearance is associated with the delayed emergence (4–8 weeks post-infection) of a CD8+ T cell response targeting multiple epitopes and exerting diverse effector functions, such as cytokine production and cytolytic activity [76]. While acute clearance of HCV leads to the development of long-lasting memory CD8+ T cells, both chimpanzees and humans remain susceptible to reinfection, indicating the formation of limited protective immunity [14,75].

Interestingly, a recent investigation into the acute HBV-specific CD8+ T cell response revealed different effector functions of CD8+ T cells targeting epitopes from the envelope, polymerase, and core proteins [77]. Distinct epitopes were linked to significantly higher expression of markers associated with memory and cytotoxicity [77]. This work sheds new light on the importance of a broad CD8 epitope response to hepatic viruses.

While CD8+ T cells are critical for viral clearance, they may also cause liver injury during acute clearance. Studies with chimpanzees and the HBV transgenic mouse model have determined that both non-cytolytic (IFN-γ) and cytolytic (perforin/granzymes) effector functions contribute to the elimination of HBV from the liver in vivo with cytolytic functions contributing to acute liver injury [78,79]. Similarly, in the NrHV mouse model, viral clearance is associated with CD8+ T cell mediated liver injury [45].

CD8+ T cells during acute-resolving HAV and HEV infection have been studied far less extensively [13]. Previous work had suggested that CD8+ T cells may not play a significant role in the clearance of HAV [73], but CD8+ T cell depletion in an Ifnar1^−/−^ HAV mouse model showed that these cells protect against HAV and limit infection-induced liver injury [40]. Additionally, a CD8+ T cell enhancing vaccine led to more rapid virus clearance with less liver damage [40]. Research into patient samples has indicated that the liver damage previously associated with an ineffective antiviral CD8+ T cell response may instead result from non-antigen specific bystander cells [80].

The role of CD8+ T cells in acute-resolving HEV infection remains poorly understood. Identifying HEV-specific CD8+ T cell epitopes has allowed for a more in-depth evaluation of these cells, finding a robust CD8+ T cell response that was significantly larger than typically seen in other hepatic infections [81]. Whether these cells are essential for HEV control, however, still needs to be evaluated. As mentioned previously, in a macaque model, HEV can be cleared in the absence of CD8+ T cells [74].

The nature and extent of the acute CD8+ T cell response to hepatitis viruses A–E is likely shaped by early events (such as specific gene expression in infected hepatocytes or T cell priming by hepatic APCs) in the liver microenvironment.

A comparison of gene expression profiles in the livers of infected chimpanzees and humans reveals common and distinct aspects of the various hepatic viruses [82,83,84,85]. In general, HCV infection induces a strong innate hepatic immune response with interferon-stimulated genes (ISG) upregulation during acute and throughout chronic infection [85,86,87,88]. Polymorphisms in the IFN-λ gene are strongly associated with HCV clearance versus persistence in humans [89,90]. HEV-infected chimpanzees upregulated most of the genes also upregulated in the context of HCV infection but to a lesser extent [82]. HAV and HBV only induce a weak hepatic innate immune response [83,84].

Overall, acute clearance of HCV, in contrast to HBV, occurs in the context of a strong ISG response [12,84,86]. Thus, how different hepatic gene expression and ISG profiles induced by these hepatic viruses are linked to a successful CD8+ T cell response remains unclear. Interestingly, a recent study showed that the IFN-λ4 variant associated with HCV persistence impairs antigen presentation in hepatocytes and attenuates the HCV-specific CD8+ T cell response in an in vitro model [91]. While these findings need to be confirmed in human patients or an animal model, they could provide a possible link between hepatic IFN-λ expression and the T cell response.

Mouse models have allowed for a much deeper examination of the CD8+ T cell priming stage in hepatic viral infections. As mentioned in the previous section, the liver contains several subsets of APCs, and T cell priming can occur directly in the liver which mostly leads to the induction of T cell tolerance [60,62]. However, while a study using an HBV mouse model highlighted the importance of extrahepatic priming [92] by eliminating antigen presentation in hepatocytes, the same researchers showed that antigen presentation in the liver is also critical for the formation of a proper acute hepatic immune response [92]. Other studies using the HBV transgenic mouse model and adoptive transfer of naïve HBV-specific T cells recently showed that priming by Kupffer cells results in the differentiation of antiviral effector CD8+ T cells, while priming by hepatocytes results in dysfunctional T cells [29]. A specific subset of Kupffer cells could reprogram T cell dysfunction induced by hepatocyte priming in an IL-2 dependent manner [93].

Work on LCMV (lymphocytic choriomeningitis virus) infection and other viral models showed that beyond priming, aggregates of myeloid cell subsets can form in the liver under inflammatory conditions and help the expansion of antiviral CD8+ T cells [94]. Ideally, studies assessing T cell priming and other early events in the formation of hepatic T cell responses would be extended to true infection models resembling acute human hepatic infections.

Interestingly, a study using the natural HEV pig model to investigate the early stages of HEV infection detected HEV antigens at high levels in various tissues prior to being found in the liver [95]. It has long been known that, despite the liver being the primary source of replication, other cell types, such as the placenta, could be infected [96]. However, this study found signs of infection starting outside the liver, suggesting that the initial immune response to the virus could be extrahepatic [95]. Given the immunosuppressive environment of the liver, this could have important implications for the priming of the successful adaptive immune response to HEV and the predominantly acute-resolving nature of this infection.

In summary, the important role of CD8+ T cells in the clearance of acute viral hepatitis is well established. However, additional research is necessary to better understand the determinates of protective CD8+ T cell immunity in the hepatic microenvironment.

### 4.2. CD4+ T Cells

CD4+ T cells play a significant role in the adaptive immune response to hepatic viral infections. Chimpanzees depleted of CD4+ T cells are incapable of clearing HCV infection [97]. These results are further supported by recent work in the NrHV mouse model which has shown that temporary depletion of CD4+ T cells prior to infection is sufficient to prevent clearance of the virus even after CD4+ T cells return to their former levels [45]. In HBV infection, CD4+ T cell depletion in chimpanzees prior to infection leads to viral persistence, while CD4+ T cell depletion during acute infection has no effect on viral clearance [71,98]. More recent studies using HBV mouse models have helped to solidify the important role of CD4+ T cells during acute infection. In fact, the capacity for clearance of HBV in mice transfected with HBV plasmids dependent on the strength of the CD4+ T cell response [99]. Additionally, successful therapeutic vaccination against HBV proteins in AAV-HBV infected or HBV transgenic mice was shown to depend on CD4+ T cell activation [100].

Analysis of blood and liver samples from chimpanzees and humans infected with HCV or HBV have revealed an association between acute clearance and a sustained CD4+ T cell response directed against a wide variety of viral epitopes (reviewed in [76,101]). Functionally, these CD4+ T cells show a strong Th1 signature with the production of IFN-γ, TNF-α, and IL-2. However, IL-21 producing CD4+ T cells and T follicular helper (Tfh) cells have also been associated with HCV and HBV clearance [76,101]. In support of these findings, in HBV mouse models the capacity to clear HBV was associated with a strong Tfh cell response [99,102].

Similar to the CD8+ T cell compartment, the role of CD4+ T cells during acute HAV and HEV has been less well studied. Infections in chimpanzees revealed that CD4+ T cells were the dominant T cell response during HAV infection and more heavily correlated with clearance than CD8+ T cells [73]. Th1 and Th2 cytokines were produced at similar levels during acute infection [73]. A study using the Ifnar1^−/−^ HAV mouse model confirmed that the absence of CD4+ T cells is linked to a lack of viral control and increased pathology [40]. The CD4+ T cells that dominated this response were found to produce IFN-γ upon restimulation in vitro [40].

HEV clearance in the absence of CD8+ T cells in rhesus macaques was associated with a strong hepatic HEV-specific CD4+ T cells response, suggesting that CD4+ T cells may also play a prominent role in this hepatic infection [74]. In addition, a study of HEV patients that either cleared or experienced liver failure after HEV infection found that successful clearance was defined by a Th1 phenotype, while an inability to clear the virus was characterized by a shift towards a Th2 response [103].

Together, human studies and animal models allowing for the manipulation of the CD4+ T cell compartment have repeatedly reinforced the importance of this T cell subset in the clearance of hepatic infections. Nevertheless, there is still a significant lack of knowledge about the exact functions of CD4+ T cell subsets in this setting. It is thought that the main function of CD4+ T cells is to provide help in the priming of a functional CD8+ T cell and B cell response. However, experimental data showing when, where, and how CD4+ T cell help might occur during acute hepatic infections are limited. It is also not well understood if and how virus-specific CD4+ T cells can be primed in the liver and how the hepatic microenvironment influences the CD4+ T cell response.

### 4.3. Regulatory T Cells

Given their immune-regulatory functions, Treg cells may be associated with reduced liver damage and the suppression of the antiviral immune response during viral clearance. For example, in a study of HAV-infected patients, more severe liver damage was associated with reduced numbers of peripheral Treg cells [104]. However, increased pathology in HAV has been associated with TNF-α secreting regulatory T cells with a Th17-like phenotype [105]. These divergent findings suggest a complex role of Treg cells in HAV. Functional regulatory T cells have also been found in the context of HEV infection [106].

Little is known about the role of Treg cells during acute HCV and HBV infection [107]. Interestingly, an examination of patients with acute-resolving HCV infection found a CD4+ T cell response more heavily skewed towards a Th1/Th17 phenotype, while patients who progressed to chronic infection showed elevated levels of Treg cells [108], indicating that Treg activity might contribute to the development of chronic infection. Changes in the balance between Th17 and Treg cells have also been associated with acute and chronic HBV infection [109].

While T cells with suppressive regulatory functions are mostly CD4+ T cells, there has been evidence of liver-primed virus-specific CD8+ T cells developing a regulatory phenotype associated with IL-10 secretion and suppression of antiviral effector T cells in a mouse model of adenoviral vector-based viral hepatitis [110]. In contrast, an analysis of IL-10 secreting CD8+ T cells in humans and mice with HBV implied that their production of anti-inflammatory cytokines enhanced rather than suppressed effector functions [111].

### 4.4. Unconventional T Cell Subsets

UTCs are enriched in the mouse and human liver and with their high functional plasticity might play antiviral, pathological, or immune-regulatory roles during acute hepatic infections [65]. Studies in HBV mouse models showed NKT cell mediated antiviral activity and liver damage [112,113,114]. HBV infection induced changes in the hepatic lipid metabolism which activated NKT cells via CD1d-TCR interactions. These activated NKT cells then contributed to the induction of the antiviral T cell response [112].

In contrast, in an examination of HEV patients, NK and NKT-like cells were found to be relatively inactive during an acute-resolving infection and showed signs of functional impairment [115]. Furthermore, recent evaluations of blood from patients with acute HCV showed that iNKT cells may be associated with acute liver damage [116]. However, using the NrHV mouse model has allowed for a more in-depth investigation of the role of hepatic iNKT cells [117]. This work suggested that hepatic IL-4 and IL-13 secreting iNKT cells may limit rather than enhance liver damage by suppressing the antiviral T cell responses [117]. Thus, functionally distinct iNKT cell subsets might be active in blood and liver during different stages of acute infection [116,117].

Few studies have addressed the role of γδ T cells and MAIT cells during acute infection. In mouse models of HBV infection, γδ T cells were found in increased numbers in the liver and actively producing IFN-γ during the first days of infection [118]. Furthermore, the transfer of hepatic CXCR3 + CXCR6 + γδ T cells from naïve mice to mice with acute HBV infection led to reduced disease and viral loads [119]. One study found that UTCs, including γδ T cells and MAIT cells, are functionally altered, both during and after the clearance of acute HCV in patients [120]. A more specific function of MAIT cells was shown in acute HAV infection. Hepatic MAIT cells showed innate-like TCR/MR1 independent cytotoxicity, which was associated with liver injury in acutely HAV-infected patients [121].

Overall, further investigation is needed to define the diverse functions of hepatic UTCs during acute viral hepatitis.

## 5. Hepatic T Cell Subsets during Chronic Viral Hepatitis

HCV and HBV cause chronic hepatic viral infections. While chronic HCV infection is characterized by stable low level viral replication [4], patients with chronic HBV infection show different phases of immune-tolerance and immune-activation associated with increases in liver damage or shifts in viral load [5]. Despite these differences, T cell responses during chronic HCV and HBV infection show significant similarities (Figure 1).

### 5.1. CD8+ T Cells

Chronic HCV and HBV infection is associated with CD8+ T cell exhaustion and dysfunction. Chronic antigen-stimulation is thought to be a major driver for T cell exhaustion and, over the last decade, significant progress has been made in identifying the phenotypical, functional, and transcriptomic characteristics of exhausted CD8+ T cell subsets in chronic viral hepatitis. (reviewed in [14,76]). In summary, antigen-specific CD8+ T cells are present during chronic infection, but they have been found in much lower numbers and tend to show high expression of various inhibitory receptors, such as PD-1, Tim-3, 2B4, or CTLA-4, and the transcription factor TOX, an essential driver of T cell exhaustion. They also exhibit weaker effector functions which leads to the inability to clear the persisting infection [14,76].

Virus-specific CD8+ T cells are initially generated during the acute phase in patients that progress to chronic HCV and HBV infection [76]. However, even early during the acute phase, CD8+ T cells from these patients show development towards a dysfunctional phenotype, associated with reduced antiviral effector functions and metabolic dysregulation [122]. The mechanisms of this process are not well understood but a lack of CD4+ T cell help (see next section) is thought to play an important role. HCV is a rapidly mutating RNA virus and viral-escape from CD8+ T cell epitopes has been shown to be another key factor in the development of HCV persistence in chimpanzees and humans [123].

Recent studies using the HCV-related hepacivirus rat model in which chronic infection is established have shown that dysfunctional virus-specific CD8+ T cells without effector functions are primed during the acute phase [124]. Testing potential HCV vaccine vectors in this model revealed that vaccine induced immunity is dependent on both CD4+ and CD8+ T cells [47].

Once HCV or HBV persistence is established, human virus-specific CD8+ T cells consist of a heterogeneous population showing varying levels of exhaustion markers and varying capacities to perform effector functions, especially during HBV infection, as recent studies have shown [125,126,127]. This heterogeneity has partly been explained by differential phenotypes of CD8+ T cells targeting epitopes in different viral proteins such as HBV core vs. HBV polymerase. Even CD8 T cells targeting different epitopes in the same viral protein, as has been shown for HCV NS3, can exhibit a distinct phenotype [77,128,129]. The cause of these epitope-based phenotypical and functional differences is not entirely understood; however, recent work on human liver parenchyma and in vitro systems may provide a possible mechanism. It was shown that hepatocytes exhibit a differential presentation of HBV nucleocapsid and envelope specific epitopes under different immunological conditions, such as the presence of IFN-γ or the level of viral protein production [130].

The development of the HCV DAA cure has provided a unique opportunity to assess CD8+ T cell function after clearance of a chronic human infection [131]. Recent work on this topic showed that certain signatures of peripheral and hepatic T cell dysfunction still appear to persist, although to a lesser extent, even after clearance of HCV [76,131]. These findings indicate that the removal of chronic antigen-stimulation is not sufficient to completely revert T cell exhaustion.

How the hepatic microenvironment contributes to the establishment of chronic infection and T cell dysfunction during the acute phase is not well understood. The tolerogenic immune-suppressive nature of the liver, with high expression of PDL-1 or IL-10 by KCs, LSECs, or HSC, might play a role in this process [16,60]. Furthermore, direct priming of CD8+ T cells by hepatocytes can lead to cell death and dysfunctionality [29,132]. An HBV transgenic mouse model modified through parabiosis with a wild-type mouse has helped provide insights into chronic HBV infection [133]. This model highlighted the capacity for a strong CD8+ T cell response in chronic HBV infection and the importance of extrahepatic priming of CD8+ T cells in maintaining CD8+ T cells in chronic infection [133].

A distinct mechanism of hepatic control of the antiviral T cell response was recently revealed using the LCMV mouse model. During chronic infection, type 1 interferon disrupts the urea cycle in hepatocytes, leading to altered serum levels of arginine and ornithine [134]. The altered metabolism contributed to the suppression of virus-specific CD8+ T cells and limited liver pathology [134]. However, whether this mechanism also plays a role in an exclusive hepatotropic infection still needs to be confirmed. It is conceivable that other metabolic functions of the liver may also influence hepatic T cell immunity. For example, the liver is the main site of cholesterol metabolism and hepatitis viruses exploit host lipids and cholesterol for their life cycles [135]. Altered availability of cholesterol during infection may influence the antiviral immune response [136]. Highlighting this aspect of liver immunology is the recent evidence that manipulating the level and distribution of cholesterol in T cells and dendritic cell membranes can influence their level of anti-HBV activity [137,138].

Unlike acute hepatic viral infections, the greater availability of liver samples from chronically infected patients has allowed for a more in-depth analysis of the human hepatic T cell landscape in established chronic viral hepatitis [139,140]. While hepatic virus-specific CD8+ T cells limit chronic HCV or HBV replication, they also contribute to progressive liver damage [30,141]. Distinct immune-regulatory mechanisms can control this CD8+ T cells response, including suppression by myeloid derived suppressor cells (MDSC) or NK cell mediated CD8+ T cell apoptosis [141]. Recent studies using scRNA-seq. and other multiparametric analysis of human liver samples revealed specific immune signatures associated with antiviral activity or liver pathology during different stages of chronic HBV infection [142,143,144].

Analysis of intrahepatic T cells has also helped to identify new subsets of CD8 T cells in hepatic viral infections. CD8+ T cells expressing IL-10 or IL-17 have been seen in liver biopsies of chronically HCV and HBV infected individuals [145,146,147,148]. IL-10 producing cells were associated with CD8+ T cell suppression in chronic infection. This contrasts with acute HBV infection, where IL-10 producing CD8+ T cells appeared to have an inflammatory rather than suppressive effect [111]. Thus, the potential functions of hepatic IL-10+ and IL-17+ CD8+ T cell subsets need further investigation.

In addition, Trm cells expressing CD69, CXCR6, and CD103 were detected in HBV and HCV infected livers [149,150,151]. In HBV infection these cells were virus-specific, expressed high levels of IL-2 but showed reduced cytotoxicity, and were preferentially enriched in patients with lower viral load [149]. In contrast, a population of non-virus specific liver-resident bystander CD8+ T cells contributes to liver damage through cytotoxic functions in chronic HBV infection [152]. The activation of cytotoxic bystander CD8+ T cells has also been implicated in mediating liver pathology in HCV and HDV infection [153,154]. HDV is a coinfection of chronic HBV and HBV/HDV coinfection is associated with the most severe progression of liver disease. However, HDV-specific hepatic T cell immunity has been less studied so far [155].

In conclusion, extensive research has resulted in a profound understanding of CD8+ T cell responses during chronic viral hepatitis. Future studies are required to elucidate the mechanisms of T cell dysfunction in the hepatic microenvironment during the acute phase and the subsequent establishment of viral persistence. A better identification of beneficial versus pathological roles of distinct hepatic CD8+ T cell subsets is also needed.

### 5.2. CD4+ T Cells

During acute HCV infection, patients with a self-limiting infection and those who progress to chronicity initially have a comparable functional CD4+ T cell response targeting multiple viral epitopes [156,157]. However, progression to chronic infection is associated with the rapid disappearance of these cells [156]. The timing and the mechanisms that lead to the failure of CD4+ T cell responses in those patients who develop a chronic infection is still little understood. This is an important topic to address since, as mentioned earlier, CD4+ T cell failure and a lack of CD4+ T cell help for CD8+ T cells is likely a major contributor to CD8+ T cell dysfunction and the development of chronic infection.

While reduced in numbers, there is still a detectable antigen-specific CD4+ T cell response in the blood and liver to a variety of epitopes throughout the course of chronic HCV and HBV infection [158,159]. These cells exhibit phenotypical and functional dysfunction characterized by limited proliferation, a weak Th1 cytokine response, and the upregulation of inhibitory markers [158,159]. Tfh cells also show a dysfunctional phenotype and a reduced production of IL-21 that, unexpectedly, does not impair B cell responses [160,161]. Mouse models of HBV infection showed that stimulation with IL-33 increased Tfh cell activity and reduced HBV viremia in vivo [162].

In general, CD4+ T cell exhaustion in established chronic HCV and HBV infection is less well studied than CD8+ T cell exhaustion. Recent work has highlighted a potential role of telomere and DNA damage in CD4+ T cell impairment [163,164].

Similar to the CD8+ T cell compartment, DAA-mediated clearance of chronic HCV infection can reverse some but not all aspects of exhausted dysfunctional CD4 T cell phenotypes [76]. Interestingly, most aspects of Tfh cell dysfunction in HCV-infected individuals can be corrected upon HCV cure [165,166].

CD4+ T cells may also play pathological roles in chronic infection. A recent characterization of human liver tissue identified multiple populations of tissue-resident and circulating CD4+ T cells [167]. A distinct CD4+ T cells subset prone to IL-4 production correlated with necroinflammatory scores in chronic HBV infection, suggesting a role in promoting liver damage [167]. In addition, virus-specific CD4+ T cells producing TNF-α are associated with liver damage in chronic HBV [168]. Th17 cell levels are increased in both HCV and HBV infection and may contribute to liver disease progression and fibrogenesis by mechanisms that include the activation of HSC and the increased production of profibrotic factors [169].

### 5.3. Regulatory T Cells

During chronic viral infection Treg cell mediated suppression of the antiviral T cell response may limit liver pathology, but it can also contribute to T cell dysfunction.

Analysis of intrahepatic and peripheral T cells during chronic HBV and HCV infection has indicated an increased proportion of Treg cells [107]. In addition, the expression of specific viral antigens, such as HCV p7 or HBV envelope, by infected hepatocytes may promote the expansion of Foxp3 + CD25 + CD4+ Treg cells in the liver [170,171,172]. A recent transcriptional analysis of the livers of chronically HBV-infected patients has shown that general biomarkers for liver damage, such as alanine aminotransferase (ALT), have been associated with increased levels of Treg cells [142]. Furthermore, in an HBV mouse model Treg cell mediated suppression of hepatic Tfh cell responses leads to persistent HBV infection [99]. Overall, while some research shows a correlation between Treg cell numbers and limited liver damage, other studies show that Treg cells are associated with suppression of the antiviral T cell response and viral persistence [107]. These findings indicate that Treg cell actions indeed result in a dual outcome which is likely dependent on the specific hepatic immunological environment of each infected individual. Future research needs to define the exact role and mechanisms of Treg cells during chronic viral hepatitis. In recent years it has also become evident that Treg cell functions extend beyond suppression of immune responses and that these also play an important role in tissue repair and homeostasis [173]. Whether or not these Treg cell mediated functions play a role in acute or chronic viral hepatitis has not been studied yet.

### 5.4. Unconventional T Cell Subsets

Among UTC subsets, MAIT cells have been most extensively characterized in the context of chronic viral hepatitis, while data on NKT cells and γδ T cells are limited and partially contradictory [68,174].

MAIT cells have been found at decreased levels in the livers and blood of chronically HCV-infected individuals [175,176,177] and exhibit an exhausted phenotype. Interestingly, MAIT cell levels or functions could be restored in the liver but not the peripheral blood of individuals who were cured with DAAs [175,177]. Enhanced cytokine activity, particularly granzyme B activity, has been seen in intrahepatic MAIT cells of HCV-infected individuals [175,178]. This shift in effector functions may be linked to increased IL-18 production in the HCV-infected liver [178]. Furthermore, in vitro tests with MAIT cells suggested that these cells could suppress HCV replication [178].

Similar to HCV, several studies reported reduced MAIT cell numbers associated with an activated and exhausted phenotype in chronic HBV and HDV infection [179,180,181,182]. One study found no evidence of HBV-related peripheral MAIT cell loss. In the same study, however, liver samples from individuals receiving nucleoside analog therapy showed higher MAIT cell levels as compared to untreated controls [183], indicating that, like in HCV, antiviral treatment might rescue intrahepatic MAIT cells. MAIT cells showed cytolytic MR1 dependent activity against HBV-transfected hepatocytes, indicating antiviral activity of these cells [181].

Overall, activated but exhausted hepatic MAIT cells with enhanced cytolytic activity might contribute to liver damage but failed viral control in chronic HCV and HBV infection.

Further evaluation of the exact role of UTC subsets, especially NKT and γδ T cells [68], in the hepatic microenvironment during chronic viral hepatitis is warranted. The significant functional and phenotypical plasticity of UTCs may explain divergent findings from different experimental setups.

## 6. Conclusions

Extensive research has resulted in significant insights into the important role of T cell subsets in mediating viral clearance during acute hepatic viral infections as well as mechanisms of T cell dysfunction during chronic infection and beneficial versus pathological roles of specific T cell subsets. While some mechanisms of T cell action and regulation in the hepatic microenvironment have been identified, there is still a lack of understanding of how this specific immunological niche and its cell interactions contribute to the generation of functional T cell responses and viral clearance versus T cell dysfunction, viral persistence, and liver pathology (Figure 1). There is also still a gap in knowledge about the hepatic mechanisms of memory T cell formation during acute-resolving infection and the phenotypical and functional characteristics of memory T cell subsets that provide protection from secondary infections. Gaining these basic insights will be important to guide the advance of successful strategies for prophylactic and therapeutic vaccines for hepatotropic viruses. Technological advances in the analysis of rare human liver tissue samples and the development of new true infection models of hepatic viruses, such as the HCV-related rodent hepacivirus rat and mouse models, should help to answer more antiviral liver immunology questions in future studies.

## Data Availability

Not applicable.

## Supplementary Materials

The following supporting information can be downloaded at: https://www.mdpi.com/article/10.3390/vaccines11030681/s1, Table S1: Rodent models for the study of T cell responses to hepatitis A–E. All mouse models are based on the C57BL/6 or Balb/c backgrounds.
