# Supplementary material for: Studying T Cell Responses to Hepatotropic Viruses in the Liver Microenvironment
Source: Vaccines (Basel). 2023 Mar 17;11(3):681. doi: 10.3390/vaccines11030681 (PMC10056334; doi:10.3390/vaccines11030681)
Supplement: Supplementary file 1 [file vaccines-11-00681-s001.zip › vaccines-2254877-supplementary.pdf]

Virus	Currently available rodent models for T cell studies	Characteristics	T cell responses	Caveats	References
**HAV**	***Immune-compromised infection models***<br>1) Ifnar1$^{-/-}$ mouse model	1) Acute HAV infection with a steady decline of viral load over 2 months; acute liver injury with a peak one-week post infection	1) HAV-specific T cell responses are generated during infection; T cells protect against infection and liver injury	The lack of type I interferon signaling in this mouse model may impair T cell responses	[39,40]
**HBV**	***Immune-competent non-infection models***<br>1) HBV transgenic mouse model<br>2) Hydrodynamic delivery of HBV genomes into the liver of mice<br>3) AdV or AAV viral vector delivery of HBV genomes into the liver mice	1) Continuous production of infectious HBV particles; no liver pathology<br>2) Transient or persistent (up to 6 months) HBV particle production; development of liver fibrosis; different outcomes depending on methods and delivery vectors<br>3) Transient or persistent HBV particle production; development of liver pathology and fibrosis; different outcomes depending on methods and delivery vectors	1) Mice are immunological tolerant to HBV; adoptive transfer of HBV-specific T cells from naïve donor mice allows T cell studies<br>2) The generation of antiviral as well as tolerogenic T cell responses to HBV antigens have been reported<br>3) The generation of antiviral as well as tolerogenic T cell responses to HBV antigens have been reported	No natural infection; HBV delivery vectors may influence the immune response	1) [26-29]<br>2) [31,34]<br>3) [32,33, 35-37]
**HCV**	***Immune-competent surrogate infection models***<br>1) HCV-related rodent hepacivirus mouse model<br>2) HCV-related rodent hepacivirus rat model	1) Acute hepatotropic infection which resolves in 3-5 weeks; acute liver injury with a peak 14 days post infection<br>2) Persistent hepatotropic infection; chronic liver inflammation	1) Virus-specific T cell responses are generated during infection; viral clearance is T cell dependent; T cell mediated liver injury<br>2) Dysfunctional T cells are primed during persistent infection	Significant genetic divergence between rodent hepaciviruses and HCV	1) [45]<br>2) [46,124]
**HEV**	no reliable model available				

Table S1. Rodent models for the study of T cell responses to hepatitis A-E. All mouse models are based on the C57BL/6 or Balb/c backgrounds.
